# Antibiotic susceptibility patterns of *Pseudomonas aeruginosa* at a tertiary care hospital in Gujarat, India

**DOI:** 10.4103/0253-7613.44156

**Published:** 2008-10

**Authors:** Viren A. Javiya, Somsuvra B. Ghatak, Kamlesh R. Patel, Jagruti A. Patel

**Affiliations:** Shri Swaminarayan Sanskar Pharmacy College, Zundal, Ahmadabad, India; 1 Department of Pharmacology, Institute of Pharmacy, Nirma University of Science and Technology, Ahmedabad, India; 2 Rajasthan Hospitals, Ahmadabad, India

**Keywords:** Antimicrobial susceptibility, carbapenem sensitivity, combination antibiotics, disk diffusion technique, *Pseudomonas aeruginosa*

## Abstract

**Objectives:**

The present study was undertaken to assess the antibiotic susceptibility patterns of *Pseudomonas aeruginosa* at a tertiary care hospital in Gujarat, India. Due to significant changes in microbial genetic ecology, as a result of indiscriminate use of anti-microbials, the spread of anti-microbial resistance is now a global problem.

**Materials and Methods:**

Out of 276 culture positive samples, 56 samples of *Pseudomonas aeruginosa* were examined and 10 different types of specimen were collected. Microbial sensitivity testing was done using disk diffusion test with *Pseudomonas species* NCTC 10662, as per CLSI guidelines.

**Results:**

The highest number of *Pseudomonas infections* was found in urine, followed by pus and sputum. Pseudomonas species demonstrated marked resistance against monotherapy of penicillins, cephalosporins, fluoroquinolones, tetracyclines and macrolides. Only combination drugs like Ticarcillin + Clavulanic acid, Piperacillin + Tazobactum, Cefoperazone + Sulbactum, Cefotaxime + Sulbactum, Ceftriaxome + Sulbactum and monotherapy of amikacin showed higher sensitivity to Pseudomonas infections; however, the maximum sensitivity was shown by the Carbapenems.

**Conclusion:**

From the present study, we conclude that urinary tract infection was the most common hospital acquired infection. Also, co-administration of β -lactamase inhibitors markedly expanded the anti-microbial sensitivity of semi-synthetic penicillins and cephalosporins. The aminoglycoside group of antibiotics - amikacin - demonstrated maximum sensitivity against pseudomonas species. Therefore, use of amikacin should be restricted to severe nosocomial infections, in order to avoid rapid emergence of resistant strains. Periodic susceptibility testing should be carried out over a period of two to three years, to detect the resistance trends. Also, a rational strategy on the limited and prudent use of anti-Pseudomonal agents is urgently required.

## Introduction

Multiple antibiotic resistance in bacterial populations is a pervasive and growing clinical problem, which is recognized as a threat to public health. Hence, there is a need to conduct area-specific monitoring studies to profile different pathogens responsible for specific infections and their resistance patterns, so as to generate data that would help clinicians to choose the correct empirical treatment.

*Pseudomonas aeruginosa (P. aeruginosa)* is an epitome of opportunistic nosocomial pathogen, which causes a wide spectrum of infections and leads to substantial morbidity in immuno-compromised patients. Despite therapy, the mortality due to nosocomial pseudomonal pneumonia is approximately 70%.[1] Unfortunately, *P. aeruginosa* demonstrates resistance to multiple antibiotics, thereby jeopardizing the selection of appropriate treatment.[2] Therefore, the present study was undertaken to find out the antibiotic susceptibility patterns of pathogenic isolates of *Pseudomonas aeruginosa* from various specimens of hospital acquired infections (HAI).

## Materials and Methods

Our study group comprised of samples, which were clinically suspected cases of bacterial infections. The project was undertaken at Rajasthan Hospital, Ahmedabad, India, between January and April 2006.

Five hundred and seventy two non-duplicate isolates were taken (i.e. multiple isolates of the same species from the same patient were excluded). Two hundred and seventy six samples obtained from sputum, endo-tracheal Tract secretion, broncho-alveolar lavage, blood, urine, body tissues, pus, semen, cerebro-spinal fluid (CSF), and body fluids (peritoneal fluid) and costal bronchial Secretions (CBS) reported the presence of bacterial infection.

Identification of all isolates was carried out by a positive reaction to oxidase and production of pyocyanin.[3] Culture examination was carried out using Nutrient agar and MacConkey's medium, followed by inoculation by four flame streak method.

Antibiotic susceptibility was confirmed by disk diffusion technique on Muller-Hinton medium (Becton Dickinson Microbiological Systems, Cockysville, MD), performed according to the Clinical Laboratory Standard Institute (CLSI) guidelines.[3] Paper disks (Hi-media, Mumbai) were impregnated with antibiotics (Sigma Chemical Co., St. Louis, Mo.): Penicillins: ampicillin (10mcg), amoxycillin (20mcg), ticarcillin (75mcg), piperacillin (100mcg); cephalosporins: cephalexin (30 mcg), cefuroxime (30mcg), cefazolin (30mcg), cefotaxime (30mcg), ceftriaxone (30mcg), ceftazidime (30mcg), ceftizoxime (30mcg), cefoperazone (75 mcg), cefpodoxime 10 mcg), cefdinir (5 mcg), cefepime (30 mcg); carbepenems: imipenem (10mcg), meropenem (10 mcg); monobactums: aztreonem (30 mcg); combinations: ampicillin + sulbactum (10/10 mcg), amoxycillin + clavulinic acid (20/10 mcg), piperacillin + tazobactum (100/10 mcg), ticarcillin + clavulinic acid (75/10 mcg), cefoperazone + sulbactum (75/10 mcg), cefotaxime + sulbactum (30/10 mcg), ceftriaxone + sulbactum (30/10 mcg); Aminoglycosides: gentamicin (10 mcg), tobramycin (10 mcg), amikacin (30 mcg), netilmicin (30 mcg); quinolones: ciprofloxacin (5mcg), ofloxacin (5mcg), levofloxacin (5mcg), gatifloxacin (5mcg); tetracyclines: doxycycline (30mcg), minocycline (30mcg); macrolides: azithromycin (15mcg) and miscellaneous: chloramphenicol (30 mcg) respectively. They were incubated overnight at 37°C in 5-10% CO_2_ enriched environment (candle jar). The diameter of the zone of inhibition was measured and compared to that of standard strain and the results were interpreted as sensitive, intermediate resistant or resistant, based on CLSI guidelines.[4] The category “susceptible” was defined as identification of a strain as susceptible by the disk diffusion method. Quality control strains of *Pseudomonas species* NCTC-10662 was used to validate the results of the antimicrobial discs.

Susceptibility data were compared by using a Chi-square test with statistical package for the social sciences (SPSS) software for Windows, version 12. Both susceptibility and resistance were calculated as percentages with 95% confidence intervals. The analysis was performed on the cross-tabulated values of the presence of the resistant/intermediate/susceptible isolates, according to the categories of the selected variable. A *P* value of < 0.05 was considered to be statistically significant.

## Results

Of the 572 samples subjected to culture sensitivity, 276 reported presence of bacterial infection, thereby suggesting 48.25% as the occurrence level. The percentage occurrence of *Pseudomonas* in these 276 samples was only 20.28% (56 samples), of which 62.5% (i.e. 35 samples) and 37.5% (i.e. 21 samples) were reported from males and females respectively. Various specimens studied under the present investigation included urine, pus, sputum, blood, endotracheal secretions (ET), semen, catheter tip (CT), stool, body fluids and body tissues. The age- and gender-wise percentage and frequency of the pathogenic organism (*P. aeruginosa*) are mentioned in [Table 1].

**Table 1:** Age and gender wise percentage and frequency distribution of *P. aeruginosa* from specipic sites

*Age group (Years)*	*Total number of cases*	*Percentage of total case*	*Specimen site*													
			
			*Urine*		*Pus*		*Sputum*		*ET*		*Semen*		*CT*		*Tissue*	
									
			*M*	*F*	*M*	*F*	*M*	*F*	*M*	*F*	*M*	*F*	*M*	*F*	*M*	*F*
0-9	1	1.78	0	0	0	1	0	0	0	0	0	0	0	0	0	0
10-19	0	0.00	0	0	0	0	0	0	0	0	0	0	0	0	0	0
20-29	5	8.93	0	1	0	1	3	0	0	0	0	0	0	0	0	0
30-39	8	14.28	0	1	1	1	1	2	1	0	0	0	0	0	1	0
40-49	12	21.43	1	0	6	0	0	2	2	0	1	0	0	0	0	0
50-59	8	14.28	1	2	1	1	2	1	0	0	0	0	0	0	0	0
60-69	14	25.00	4	2	0	1	0	1	4	1	0	0	1	0	0	0
70-79	4	7.14	0	1	1	1	1	0	0	0	0	0	0	0	0	0
80-more	4	7.14	0	2	0	0	2	0	0	0	0	0	0	0	0	0
Total	56	100	6	9	9	6	9	6	7	1	1	0	1	0	1	0

ET = Endo-tracheal tract secretions, CT = Catheter tip, M = Male, F = Female

The majority of specimens from which *P. aeruginosa* was isolated consisted of urine, pus and sputum [Table 2]. The acid resistant penicillins such as ticarcillin and piperacillin combinations (R=23.21% and 30.36% respectively) (*P*< 0.001) had significantly greater antibacterial activity against *P. aeruginosa*, when compared to their respective monotherapies (R=67.86% and 73.21% respectively) [Fig. 1A].

**Table 2:** Frequency of specific sites from which *P. aeruginosa* was isolated

*Specimen*	*Number of Specimens*	*% of Total*
Urine	15	26.79
Pus	15	26.79
Sputum	15	26.79
ET	8	14.29
Semen	1	1.78
CT	1	1.78
Tissue	1	1.78
Total	56	100

ET: Endo-tracheal tract secretions, CT: Catheter tip

**Figure 1a:**
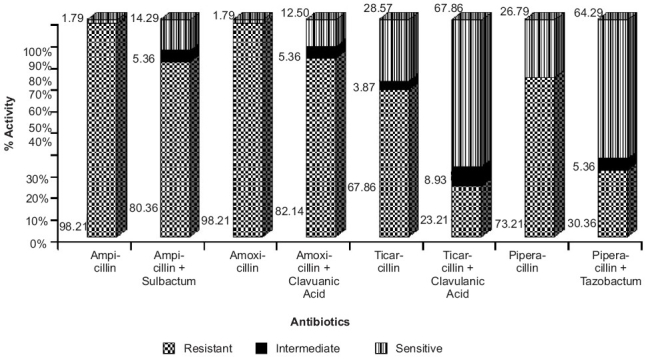
Antibiotic resistance patterns of *P. aeruginosa* against penicillin group of antibiotics. Ampicillin, amoxicillin, ticarcillin and piperacillin in combination with sulbactum, clavulanic acid and tazobactum demonstrated significantly higher antibacterial activity (P≤0.001, Chi-square test) against *P. aeruginosa* when compared to the monotherapy of respective antibiotics

A similar scenario was observed for ampicillin and amoxicillin, where the combination therapy with sulbactum and clavulanic acid (*P*< 0.001) demonstrated significantly higher antibacterial activity against *P. aeruginosa*, when compared to their respective monotherapies (R=98.21% in both the cases) [Figure 1B]. The organism showed remarkable resistance against cephalosporin group of antibiotics, ranging from 67.86% for ceftazidime to 94.64% for cephalexin [Table 3]. But the extended-spectrum penicillins and the third generation cephalosporins, in combination with sulbactum, tazobactum and clavulanic acid showed a significant decrease in resistance to *P. aeruginosa*.

**Figure 1b:**
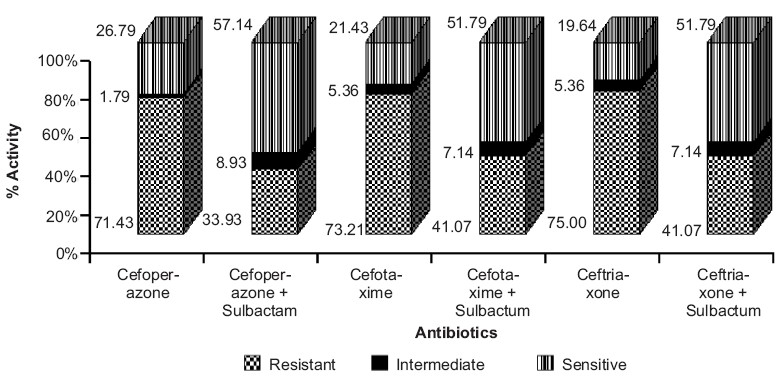
Antibiotic resistance patterns of *P. aeruginosa* against cephalosporin group of antibiotics. Cefoperazone, cefotaxime and ceftriaxone in combination with sulbactum demonstrated significantly higher antibacterial activity (*P*≤0.001, Chi-square test) when compared to monotherapy of respective antibiotics

**Table 3:** Antibiotic resistance pattern of *P. aeruginosa* against cephalosporin group of antibiotics

*Cephalosporins*	*Sensitive*	*Intermediate*	*Resistant*
Cephalexin	5.36	0	94.64
Cefazolin	12.5	3.57	83.93
Cefuroxime	7.14	0	92.86
Cefoperazone	26.79	1.79	71.43
Cefpodoxime	23.21	0	76.79
Cefdinir	23.21	0	76.79
Ceftazidime	32.14	0	67.86
Ceftriaxone	19.64	5.36	75
Ceftizoxime	32.21	0	76.79
Cefotaxime	21.43	5.36	73.21
Cefepime	30.36	0	69.64

Values shown are percentage of total

A notable observation was that piperacillin and ticarcillin combinations demonstrated better antibacterial activity, as compared to cephalosporin combinations (*P*< 0.001) [Figure 1A and B]. We observed that *P. aeruginosa* was highly sensitive to the carbapenem group of antibiotics like imipenem (78.57%) and meropenem (69.64%), while aztreonem showed 71.43% resistance (*P*< 0.001) [Figure 1C]. On the other hand, aminoglycosides, fluoroquinolones, tetracyclines, macrolides and chloramphenicol did not demonstrate statistically significant susceptibility patterns (*P*>0.05).

**Figure 1c:**
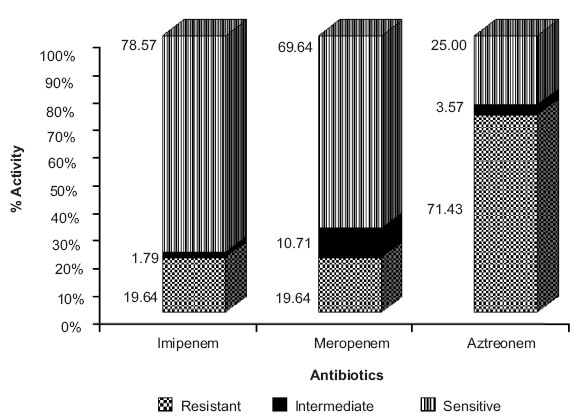
Antibiotic resistance patterns of *P. aeruginosa* against carbapenem group of antibiotics. Imipenem and meropenem demonstrated significantly higher antibacterial activity (*P*≤0.001, Chi-square test) when compared to aztreonem

In the present study, sensitivity of *P. aeruginosa* was in the 32-48% range for aminoglycosides, while for fluoroquinolones, susceptibility was found to be in the 26-37% range [Table 4]. For tetracyclines, macrolides and chloramphenicol, resistance was found to range between 75 and 91% [Table 4]. All antibiotics used as monotherapy in the present study did not demonstrate statistically significant susceptibility patterns (*P*>0.05), with the exception of imipenem and meropenem.

**Table 4:** Antibiotic resistance patterns of *P. aeruginosa* against different group of antibiotics

Class of drugs	Sensitive	Intermediate	Resistant
Aminoglycosides			
Gentamicin	32.14	0	67.86
Tobramycin	32.14	1.79	66.07
Netilmycin	33.93	5.36	60.71
Amikacin	48.21	1.79	50
Fluoroquinolones			
Ciprofloxacin	26.79	3.57	69.64
Ofloxacin	26.79	3.57	69.64
Levofloxacin	35.71	1.79	62.5
Gatifloxacin	37.5	0	62.5
Tetracyclines			
Doxycycline	8.93	0	91.07
Minocycline	10.71	1.79	87.5
Macrolides			
Azithromycin	8.93	5.36	85.71
Miscellaneous			
Chloramphenicol	22	3	75

Values shown are percentage of total; (n = 56 cases)

## Discussion

*P. aeruginosa* is inherently resistant to many antimicrobial agents, mainly due to the synergy between multi-drug efflux system or a type1 AmpC β-lactamase and low outer membrane permeability.[5–7] The age- and sex-wise distribution of patients diagnosed with infection followed the natural epidemiological pattern.[8] Out of 56 culture positive specimens isolated, 26.78% were urine samples, indicating that urinary tract infection (UTI) is the most common HAI[9] [Table 2]. It is one of the most important causes of morbidity in the general population and is the second most common cause of hospital visits.[10,11] The incidence of UTI is greater in women than men, which may be either due to anatomical predisposition or urolithial mucosal adherence to mucopolysaccharide lining or other host factors.[9,12] Concurrent administration of a β-lactamase inhibitor such as clavulanate or sulbactum markedly expands the spectrum of activity of acid resistant penicillins like ticarcillin and piperacillin. The dose as well as the incidence of toxicity were subsequently reduced with semi-synthetic penicillins like ticarcillin, which makes it the preferred ureidopenicillin against *P. aeruginosa* infections. Our results are in corroboration with the one reported by other workers,[11] so much so that the overall resistance to various generations of cephalosporins was high on account of the production of extended spectrum β-lactamses (ESBLs) by the bacteria involved.[13]

In our study, notable resistance (19.64%) to *P. aeruginosa* was observed against carbapenems. The resistance to carbapenems, especially in *P. aeruginosa*, results from reduced levels of drug accumulation or increased expression of pump efflux.[14,15] The resistance may also be due to the production of metallo-β-lactamases (MBL), which can be chromosomally encoded or plasmid mediated.[16] The carbapenem hydrolyzing enzyme carbapenamase may be class B-metallo β-lactamases or class D-oxacillanases or class A-clavulanic acid inhibitory enzymes.[17]

Among the aminoglycosides, amikacin has the highest sensitivity against *P. aeruginosa* [Table 4], which is in corroboration with an earlier report published from India.[18] Amikacin was designed as a poor substrate for the enzymes that bring about inactivation by phosphorylation, adenylation or acetylation, but some organisms have developed enzymes that inactivate this agent as well. Amikacin seems to be a promising therapy for Pseudomonal infection. Hence, its use should be restricted to severe nosocomial infections, in order to avoid rapid emergence of resistant strains.[19] The problem of increasing resistance to *P. aeruginosa* has limited the use of other classes of antibiotics like the fluoroquinolones, tetracyclines, macrolides and chloramphenicol.[20]

In fact, the irrational and inappropriate use of antibiotics is responsible for the development of resistance of *Pseudomonas species* to antibiotic monotherapy. Hence, there is a need to emphasize the rational use of antimicrobials and strictly adhere to the concept of “reserve drugs” to minimize the misuse of available antimicrobials. In addition, regular antimicrobial susceptibility surveillance is essential for area-wise monitoring of the resistance patterns. An effective national and state level antibiotic policy and draft guidelines should be introduced to preserve the effectiveness of antibiotics and for better patient management.

Hence, based on the observations of present study, we recommend use of either semi-synthetic penicillins like ticarcillin, piperacillin or third generation cephalosporins like cefoperazone, cefotaxime and ceftriaxone along with β-lactamase inhibitors (clavulanate or sulbactum) against *Pseudomonas species* infections, in similar hospital settings. Further, amikacin should be considered as a reserved drug for the treatment of severe nosocomial infections.
